# *miR*-*506*: a regulator of chemo-sensitivity through suppression of the RAD51-homologous recombination axis

**DOI:** 10.1186/s40880-015-0049-z

**Published:** 2015-09-14

**Authors:** Guoyan Liu, Fengxia Xue, Wei Zhang

**Affiliations:** Department of Gynecology and Obstetrics, Tianjin Medical University General Hospital, Tianjin, 300052 P.R. China; Department of Pathology, The University of Texas MD Anderson Cancer Center, Houston, TX 77030 USA

**Keywords:** MicroRNA-506, Homologous recombination, Drug sensitivity, Synthetic lethality, RAD51

## Abstract

Ovarian carcinoma is the most lethal gynecologic malignancy. Resistance to platinum is considered the major problem affecting prognosis. Our recent study established that microRNA-506 (*miR*-*506*) expression was closely associated with progression-free survival and overall survival in two independent patient cohorts totaling 598 epithelial ovarian cancer cases. Further functional study demonstrated that *miR*-*506* could augment the response to cisplatin and olaparib through targeting *RAD51* and suppressing homologous recombination in a panel of ovarian cancer cell lines. Systemic delivery of *miR*-*506* in an orthotopic ovarian cancer mouse model significantly augmented the cisplatin response, thus recapitulating the clinical observation. Therefore, *miR*-*506* plays a functionally important role in homologous recombination and has important therapeutic value for sensitizing cancer cells to chemotherapy, especially in chemo-resistant patients with attenuated expression of *miR*-*506*.

## Background

Epithelial ovarian cancer remains the most lethal gynecologic malignancy [1, 2]. The majority of cases are diagnosed at an advanced stage, with a 5-year survival rate of only 30%–40% [3]. Debulking surgery and platinum-based chemotherapy play important roles in the management of epithelial ovarian cancer, whereas acquired resistance to platinum is considered a major factor in disease relapse.

Recently, it has been reported that microRNAs (miRNAs), which are single-stranded, non-coding endogenous RNAs, play a crucial role in regulating drug resistance. Possible mechanisms in regulating drug resistance involve the regulation of cell cycle distribution, drug transport, DNA repair, epithelial-mesenchymal transition (EMT), and apoptosis pathways. Platinum-based drugs can form intra- and inter-strand adducts with DNA, which cause DNA double-strand breaks and trigger DNA damage and repair pathways. Thus, enhancement of the DNA repair system plays a major role in platinum resistance. Homologous recombination (HR) is a critical pathway for DNA double-strand break repair; cells with compromised HR machinery are highly sensitive to DNA-damaging drugs [4].

Our recent investigation of The Cancer Genome Atlas (TCGA) database network for high-grade serous ovarian carcinoma showed that microRNA-506 (*miR*-*506*) expression was associated with an increased response to therapy and prolonged progression-free survival (PFS) and overall survival (OS). This result was confirmed in another clinically annotated genomics dataset (Bagnoli) [5]. Our previous studies have demonstrated that *miR*-*506* can inhibit EMT and proliferation by targeting the snail family zinc finger 2 (SNAL2) and cyclin-dependent kinase 4/6-Forkhead box protein M1 (CDK4/6-FOXM1) axes, respectively [6–8]. After overexpressing *miR*-*506* in a panel of ovarian cancer cell lines, we analyzed the down-regulated genes via a microarray and observed a decrease in *RAD51* levels. When we used the miRNA target prediction algorithm TargetScan 6.0 (Whitehead Institute for Biomedical Research, Cambridge, MA, USA), we found that *RAD51* had one predicted binding site for *miR*-*506*. We next performed a set of functional validation experiments using reporter gene assays. We found that *miR*-*506* suppressed the reporter activity linked to the 3′- untranslated region (UTR) of *RAD51* that contains the predicted binding site. The use of control miRNA or removal of the binding site did not produce the same regulation exhibited by *miR*-*506*. These results showed that *RAD51* is a direct target of *miR*-*506*. Furthermore, the inverse association between *miR*-*506* and *RAD51* expression was confirmed in three different cohorts of clinical samples (the TCGA, Bagnoli, and Tianjin cohorts).

We next sought to understand how important the regulation of *RAD51* is to the drug response and survival of serous ovarian cancer patients. *RAD51* is known to be a critical component of the HR-mediated double-strand DNA break repair machinery, and during this process, RAD51 assembles onto single-stranded DNA as a nucleoprotein filament and catalyzes the exchange of homologous DNA sequences [9]. Because RAD51 is an integral component of the cellular DNA damage response, RAD51 suppression can sensitize cancer cells to DNA-damaging drugs [10–13]. By performing a homology-directed repair assay, we found that transfection with a *miR*-*506* mimic or treatment with small interfering RNA (siRNA) against *RAD51* significantly reduced the HR efficiency. In addition, ectopic overexpression of *miR*-*506* led to higher residual DNA damage (as shown by single-cell gel electrophoresis) and less RAD51 foci formation (as shown by immunofluorescence microscopy imaging**)** as compared with controls after cisplatin treatment, confirming the defect in HR.

HR-deficient cells are sensitive to DNA-damaging drugs and poly(ADP) ribose polymerase (PARP) inhibitors, and this effect has been termed synthetic lethality [14, 15]. Recent studies have demonstrated that breast cancer early onset 2 (*BRCA2*) mutations, and to a lesser extent breast cancer early onset 1 (*BRCA1*) mutations/methylation, are associated with improved survival and responses to therapy in serous ovarian cancer patients [16–18]. We next examined the effect of *miR*-*506* on the sensitivity to cisplatin and olaparib (a PARP inhibitor) in ovarian cancer in vitro and in vivo and found that *miR*-*506*-transfected cells were more sensitive to cisplatin or olaparib than control cells in both 3-(4,5-dimethyl-2-thiazolyl)-2,5-diphenyl-2-H-tetrazolium bromide (MTT) and clonogenic survival assays. Conversely, anti-miR-506 locked nucleic acid (LNA) transfection enhanced RAD51 expression and induced resistance to cisplatin and olaparib in cells. Moreover, the effect of *miR*-*506* on cisplatin and olaparib sensitivity was rescued by overexpressing RAD51 without its 3′-UTR, suggesting that *miR*-*506*-mediated sensitivity to cisplatin and olaparib is the result of the suppression of RAD51 expression. Consistent with the results of in vitro experiments, delivery of *miR*-*506* incorporated in 1,2-dioleoyl-sn-glycerol-3-phosphatidylcholine (DOPC) nanoliposomes effectively enhanced the effect of cisplatin and olaparib in an orthotopic ovarian cancer model.

**Fig. 1:**
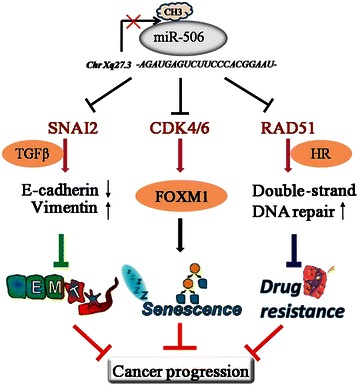
A summary of the therapeutic role of microRNA-506 (*miR*-*506*). *miR*-*506*, located on Xq27.3, is down-regulated by methylation. *miR*-*506* directly targets *RAD51*, cyclin-dependent kinase 4/6-Forkhead box protein M-1 (*CDK4/6*-*FOXM1*), and snail family zinc finger 2 (*SNAI2*), thus repressing homologous recombination (HR), promoting cellular senescence, and suppressing epithelial-mesenchymal transition (EMT), respectively. As a result, *miR*-*506* sensitizes cancer cells to chemotherapy and inhibits cell proliferation and EMT-mediated metastasis

In summary, our recent investigations have revealed that *miR*-*506* can enhance the sensitivity to cisplatin and PARP inhibitors through suppression of the RAD51-HR axis. Furthermore, nanoparticle delivery of *miR*-*506* enhanced the effects of cisplatin and olaparib in orthotopic ovarian cancer models. Thus, *miR*-*506* not only is a robust clinical marker for the chemotherapy response and survival of serous ovarian cancer patients but also has important therapeutic value in sensitizing cancer cells to chemotherapy. This new discovery shows that *miR*-*506* combined with DNA-damaging agent treatment may benefit patients with high-grade serous ovarian carcinoma. Taken together with the findings of our previous study on this small molecule, we have shown that *miR*-*506* has multiple therapeutic effects on regulating the biological behavior of cancer cells (Fig. 1).

## References

[1] Siegel RL, Miller KD, Jemal A (2015). Cancer statistics, 2015. CA Cancer J Clin.

[2] Devouassoux-Shisheboran M, Genestie C (2015). Pathobiology of ovarian carcinomas. Chin J Cancer.

[3] Cancer Genome Atlas Research Network (2011). Integrated genomic analyses of ovarian carcinoma. Nature.

[4] Roy R, Chun J, Powell SN (2011). BRCA1 and BRCA2: different roles in a common pathway of genome protection. Nat Rev Cancer.

[5] Liu G, Yang D, Rupaimoole R, Pecot CV, Sun Y, Mangala LS, et al. Augmentation of response to chemotherapy by microRNA-506 through regulation of RAD51 in serous ovarian cancers. J Natl Cancer Inst. 2015;107(7). doi:10.1093/jnci/djv108. 10.1093/jnci/djv108 PMC4554255 25995442

[6] Yang D, Sun Y, Hu L, Zheng H, Ji P, Pecot CV (2013). Integrated analyses identify a master microRNA regulatory network for the mesenchymal subtype in serous ovarian cancer. Cancer Cell.

[7] Liu G, Sun Y, Ji P, Li X, Cogdell D, Yang D (2014). MiR-506 suppresses proliferation and induces senescence by directly targeting the CDK4/6-FOXM1 axis in ovarian cancer. J Pathol.

[8] Sun Y, Guo F, Bagnoli M, Xue FX, Sun BC, Shmulevich I (2015). Key nodes of a microRNA network associated with the integrated mesenchymal subtype of high-grade serous ovarian cancer. Chin J Cancer.

[9] Baumann P, West SC (1998). Role of the human RAD51 protein in homologous recombination and double-stranded-break repair. Trends Biochem Sci.

[10] Quiros S, Roos WP, Kaina B (2011). Rad51 and BRCA2—new molecular targets for sensitizing glioma cells to alkylating anticancer drugs. PLoS One.

[11] Tsai MS, Kuo YH, Chiu YF, Su YC, Lin YW (2010). Down-regulation of Rad51 expression overcomes drug resistance to gemcitabine in human non-small-cell lung cancer cells. J Pharmacol Exp Ther.

[12] Ito M, Yamamoto S, Nimura K, Hiraoka K, Tamai K, Kaneda Y (2005). Rad51 siRNA delivered by HVJ envelope vector enhances the anti-cancer effect of cisplatin. J Gene Med.

[13] Yang Z, Waldman AS, Wyatt MD (2012). Expression and regulation of RAD51 mediate cellular responses to chemotherapeutics. Biochem Pharmacol.

[14] Farmer H, McCabe N, Lord CJ, Tutt AN, Johnson DA, Richardson TB (2005). Targeting the DNA repair defect in BRCA mutant cells as a therapeutic strategy. Nature.

[15] Chen A (2011). PARP inhibitors: its role in treatment of cancer. Chin J Cancer.

[16] Yang D, Khan S, Sun Y, Hess K, Shmulevich I, Sood AK (2011). Association of BRCA1 and BRCA2 mutations with survival, chemotherapy sensitivity, and gene mutator phenotype in patients with ovarian cancer. JAMA.

[17] Bolton KL, Chenevix-Trench G, Goh C, Sadetzki S, Ramus SJ, Karlan BY (2012). Association between BRCA1 and BRCA2 mutations and survival in women with invasive epithelial ovarian cancer. JAMA.

[18] Liu G, Yang D, Sun Y, Shmulevich I, Xue F, Sood AK (2012). Differing clinical impact of BRCA1 and BRCA2 mutations in serous ovarian cancer. Pharmacogenomics.

